# Intracellular calcium stores drive slow non-ribbon vesicle release from rod photoreceptors

**DOI:** 10.3389/fncel.2014.00020

**Published:** 2014-02-03

**Authors:** Minghui Chen, David Križaj, Wallace B. Thoreson

**Affiliations:** ^1^Department of Pharmacology and Experimental Neuroscience, University of Nebraska Medical CenterOmaha, NE, USA; ^2^Department of Ophthalmology and Visual Sciences, University of Nebraska Medical CenterOmaha, NE, USA; ^3^Department of Ophthalmology and Visual Sciences, Moran Eye Center, University of Utah School of MedicineSalt Lake City, UT, USA

**Keywords:** calcium-induced calcium release, ryanodine receptors, exocytosis, ribbon synapse, retina, synaptic vesicle, total internal reflection fluorescence microscopy

## Abstract

Rods are capable of greater slow release than cones contributing to overall slower release kinetics. Slow release in rods involves Ca^2+^-induced Ca^2+^ release (CICR). By impairing release from ribbons, we found that unlike cones where release occurs entirely at ribbon-style active zones, slow release from rods occurs mostly at ectopic, non-ribbon sites. To investigate the role of CICR in ribbon and non-ribbon release from rods, we used total internal reflection fluorescence microscopy as a tool for visualizing terminals of isolated rods loaded with fluorescent Ca^2+^ indicator dyes and synaptic vesicles loaded with dextran-conjugated pH-sensitive rhodamine. We found that rather than simply facilitating release, activation of CICR by ryanodine triggered release directly in rods, independent of plasma membrane Ca^2+^ channel activation. Ryanodine-evoked release occurred mostly at non-ribbon sites and release evoked by sustained depolarization at non-ribbon sites was mostly due to CICR. Unlike release at ribbon-style active zones, non-ribbon release did not occur at fixed locations. Fluorescence recovery after photobleaching of endoplasmic reticulum (ER)-tracker dye in rod terminals showed that ER extends continuously from synapse to soma. Release of Ca^2+^ from terminal ER by lengthy depolarization did not significantly deplete Ca^2+^ from ER in the perikaryon. Collectively, these results indicate that CICR-triggered release at non-ribbon sites is a major mechanism for maintaining vesicle release from rods and that CICR in terminals may be sustained by diffusion of Ca^2+^ through ER from other parts of the cell.

## INTRODUCTION

Light-evoked voltage changes in photoreceptor cells are transmitted to second-order retinal neurons by changing the rate of glutamate release. In cones, glutamate release is thought to occur almost exclusively at plate-like synaptic ribbons (Jackman et al., 2009; Snellman et al., 2011) where depolarization at light offset stimulates rapid release of vesicles tethered at the base of the ribbon (Bartoletti et al., 2010; Snellman et al., 2011). During maintained depolarization, the rate of release declines and is governed by the rate that vesicles replenish release sites at the base of the ribbon (Jackman et al., 2009; Snellman et al., 2011).

Although rods are also capable of fast release (Li et al., 2010), rods exhibit considerably greater slow release than cones when stimulated with long (>100 ms) depolarizing steps, contributing to overall slower release kinetics (Schnapf and Copenhagen, 1982; Cadetti et al., 2005; Rabl et al., 2005). Using total internal reflection fluorescence microscopy (TIRFM) to visualize fusion of synaptic vesicles in rods loaded with dextran-conjugated pH-sensitive rhodamine (pHrodo), many of the vesicle fusion events evoked by long depolarizing steps were found to occur at sites >1 μm from ribbons (Chen et al., 2013). Furthermore, damaging ribbons by fluorophore-assisted laser inactivation (FALI) of the ribbon protein Ribeye selectively diminished exocytotic increases in membrane capacitance evoked by short test steps but not capacitance increases evoked by longer steps (Chen et al., 2013). These results suggest that fast release from rods involves the ribbon but slow release involves non-ribbon release sites. Also consistent with contributions from non-ribbon release sites in rods are the presence of putative fusion events at non-ribbon sites revealed by electron microscopic (EM) tomography (Zampighi et al., 2011).

In addition to non-ribbon release, slow release from rods, but not cones, also involves Ca^2+^-induced Ca^2+^ release (CICR; Križaj et al., 1999; Cadetti et al., 2006; Suryanarayanan and Slaughter, 2006; Babai et al., 2010b). Blocking CICR strongly inhibited light responses in second-order neurons from mammalian and amphibian retina suggesting that CICR is essential for maintaining sustained release from rods in darkness (Cadetti et al., 2006; Suryanarayanan and Slaughter, 2006; Babai et al., 2010b). CICR has been shown to promote spontaneous (Emptage et al., 2001; Bardo et al., 2002; Simkus and Stricker, 2002) and evoked synaptic release in a number of neurons (Ludwig et al., 2002; Galante and Marty, 2003; Trueta et al., 2003). It typically does so by enhancing vesicle priming (Ludwig et al., 2002, 2005; Tobin et al., 2012), increasing vesicle mobility (Shakiryanova et al., 2007), or sensitizing vesicles to forthcoming depolarization (Llano et al., 2000; Galante and Marty, 2003). However, the unusually high Ca^2+^ sensitivity of exocytotic sensors in rods (Thoreson et al., 2004) raised the possibility that CICR may trigger release directly in rods.

In the present study, we tested the hypothesis that slow release from rods involves CICR-triggered release of vesicles at non-ribbon sites. To test this hypothesis, we combined electrophysiological recordings and TIRFM visualization of submembrane Ca^2+^ changes and vesicle fusion events (Chen et al., 2013). The results showed that most of the slow synaptic release from rods is due to vesicle fusion at non-ribbon sites triggered by CICR. The results also suggest that CICR-driven release may be sustained by Ca^2+^ ions diffusing through the endoplasmic reticulum (ER) from perikaryon to synapse.

## MATERIALS AND METHODS

### ANIMAL CARE AND USE

Aquatic tiger salamanders (*Ambystoma tigrinum*, 18–25 cm in length; Charles Sullivan Co., Nashville, TN, USA) were maintained on a 12-h light/dark cycle and killed 1–2 h after the beginning of subjective night. Salamanders were decapitated with heavy shears, the head was hemisected and the spinal cord pithed. Protocols were approved by the University of Nebraska Medical Center Institutional Animal Care and Use Committee.

### PAIRED RECORDINGS FROM RODS AND HORIZONTAL CELLS

To measure release from rods electrophysiologically, we obtained paired whole cell recordings from rods and horizontal cells in a retina slice preparation. Details of slice preparation and electrophysiological recordings are described in detail elsewhere (Van Hook and Thoreson, 2013). Briefly, retinal slices (125 μm) were placed under a water-immersion objective (60×, 1.0 NA) on an upright fixed-stage microscope (Nikon E600FN) and superfused at ~1 ml/min with an oxygenated amphibian saline solution containing (in mM): 116 NaCl, 2.5 KCl, 1.8 CaCl_2_, 0.5 MgCl_2_, 10 *N*-2-hydroxyethylpiperazine-*N′*-2-ethanesulfonic acid (HEPES), and 5 glucose (pH 7.8). Rods and horizontal cells were simultaneously voltage clamped using Optopatch (Cairn Research) and Axopatch 200B (Molecular Devices) patch-clamp amplifiers. Recording pipettes were pulled on a PP-830 vertical puller (Narishige International USA, East Meadow, NY, USA) from borosilicate glass pipettes (1.2-mm outer diameter, 0.9-mm inner diameter, with internal filament; World Precision Instruments, Sarasota, FL, USA). Pipette resistance was 12–18 MΩ. Rod pipettes were filled with (in mM): 40 cesium glutamate, 50 cesium gluconate, 9.4 tetraethylammonium chloride (TEACl), 3.5 NaCl, 1 MgCl_2_, 9.4 MgATP, 0.5 GTP, 5 ethylene glycol tetraacetic acid (EGTA), 1 reduced glutathione, 1 6-hydroxy-2,5,7,8-tetramethylchroman-2-carboxylic acid (Trolox), 10 HEPES (pH 7.2). Horizontal cell pipettes contained (in mM): 90 cesium gluconate, 10 TEACl, 1 CaCl_2_, 3.5 NaCl, 1 MgCl_2_, 9.4 MgATP, 0.5 GTP, 5 EGTA, 10 HEPES (pH 7.2). Unless otherwise specified, reagents were obtained from Sigma-Aldrich Chemicals (St. Louis, MO, USA). Currents were acquired and analyzed using pClamp 9.2 software with Digidata 1322 interface (Molecular Devices).

To selectively damage ribbons, we used FALI with a fluorescein-conjugated Ribeye-binding peptide (80 μM) added to the presynaptic patch pipette solution. The Ribeye-binding peptide (EQTVPVDLSVARPR) contains a PXDLS sequence that binds selectively to the C-terminal binding protein (CtBP) domain of Ribeye (Zenisek et al., 2004). After waiting >10 min for diffusion into the cell, the peptide was bleached by 50 s exposure to 488-nm light from an Ar/Kr laser delivered through a laser confocal scan head (PerkinElmer Ultraview LCI, Waltham, MA, USA) mounted to an upright, fixed-stage microscope (Nikon E600FN; Snellman et al., 2011). Excitation of the fluorescein moiety by 488 nm laser light generates singlet oxygen producing half-maximal damage within ~40 Å of the fluorophore (Hoffman-Kim et al., 2007). As a control, we used a scrambled version of the same fluorescein-conjugated peptide. Ribeye-binding peptide or the scrambled control peptide were added to the pipette solution together with the anti-oxidants reduced glutathione (1 mM) and 6-hydroxy-2,5,7,8-tetramethylchroman-2-carboxylic acid (Trolox, 1 mM).

To block synaptic vesicle release, botulinum toxin E light chain (500 nM), which cleaves the SNARE (soluble *N*-ethylmaleimide-sensitive factor attachment protein receptor) proteins SNAP-23 and SNAP-25, was added to the presynaptic patch pipette solution in some experiments.

### LOADING SYNAPTIC VESICLES WITH DEXTRAN-CONJUGATED pHrodo

Synaptic vesicles were visualized by TIRFM as described previously (Chen et al., 2013). Briefly, retinas were isolated and incubated with a dextran (10,000 MW)-conjugated, pH-sensitive form of rhodamine, pHrodo (500 μg/ml, Invitrogen, Grand Island, NY, USA) in amphibian saline at 20°C. During dissection and dye incubation, retinas were maintained in darkness using night vision goggles (Nitemate NAV3, Litton Industries, Tempe, AZ, USA). The depolarized membrane potential of rods in darkness stimulates continuous release of vesicles, followed by endocytosis and uptake of pHrodo. Dextran-conjugated pHrodo is water-soluble and its fluorescence increases in the acidic interior of synaptic vesicles. Fluorescence of pHrodo declines considerably at an extracellular pH of 7.8. After loading vesicles, retinas were exposed to light to hyperpolarize the rods and placed in a Ca^2+^-free amphibian saline to inhibit further exocytosis. For some experiments, we used 30-min incubation to load a large portion of the vesicle population. For other experiments, we used a short incubation time (3 min) to load 1–3% of the vesicle pool (Chen et al., 2013) and thereby visualize individual vesicles. Evidence that fluorescent organelles labeled by the latter approach were individual synaptic vesicles include the findings that they matched the diffraction-limited size of 40 nm fluorescent microspheres; fluorescence of pHrodo-loaded organelles disappeared upon depolarization with rapid kinetics matching exocytosis; and their depolarization-evoked disappearance was blocked by inhibiting Ca^2+^ channels with Co^2^^+^ (Chen et al., 2013). Fusion of individual vesicles can be distinguished from vesicle departure by the much faster decline in fluorescence following vesicle fusion compared to the slower fluorescence changes that accompany vesicle approach or departure from the membrane (Chen et al., 2013). Fusion events were identified by the following criteria: fluorescence must decline by 60% within two frames (82 ms) after the peak and exhibit a total decrease of >90% (Chen et al., 2013).

### PHOTORECEPTOR ISOLATION

After loading with pHrodo, retinas were digested by incubation with papain (30 U/ml, Worthington, Lakewood, NJ, USA) plus cysteine (0.2 mg/ml) in Ca^2+^-free amphibian saline solution for 35 min at ~20°C. After papain treatment, the tissue was washed in ice cold, Ca^2+^-free amphibian saline containing 1% bovine serum albumin and DNase (1 mg/ml, Worthington) followed by two additional washes in ice-cold, Ca^2+^-free saline. A piece of retina was then triturated with a fire-polished Pasteur pipette and the cell suspension transferred onto 1.78 refractive index glass cover slips (Olympus, Center Valley, PA, USA) coated with Cell-Tak (3.5 μg/cm^2^, BD Biosciences, San Jose, CA, USA). After letting cells settle and adhere for 30 min, they were superfused with oxygenated amphibian saline solution at 20°C. Rods were identified by their characteristic morphology. Cones and sometimes bipolar cells were also loaded with pHrodo but not examined in this study. Light-sensitive outer segments of rods were typically lost during trituration.

### CALCIUM IMAGING

Ca^2+^ channels are clustered beneath ribbons (Nachman-Clewner et al., 1999; Tom Dieck et al., 2005) and so sites of focal Ca^2+^ entry evoked by brief depolarizing steps co-localize with ribbons labeled with fluorescently tagged Ribeye-binding peptides (Choi et al., 2008; Mercer and Thoreson, 2011; Chen et al., 2013). This allows sites of focal Ca^2+^ entry to be used as an indication of ribbon location. Ribbons labeled by fluorescently conjugated Ribeye-binding peptides were typically not visible by TIRFM in rods, perhaps because their location atop the arciform density places them outside the evanescent field of illumination (Chen et al., 2013). To image Ca^2+^ entry sites, fluo-5F (100 μM, *K*_d_ = 2.3 μM, Invitrogen) was added to the pipette solution. The sites of peak fluorescence increases evoked by 50-ms depolarizing steps from -70 to -10 mV with Δ*F*/*F* > 0.5 were defined as Ca^2+^ entry sites. Different focal Ca^2+^ entry sites were defined as separate ribbons if they showed distinct peaks separated by ≥500 nm (Chen et al., 2013).

Endoplasmic reticulum Ca^2+^ levels are quite high (60–400 μM; Michalak et al., 2002). To visualize ER Ca^2+^ stores, isolated rods were therefore incubated with a low affinity Ca^2+^ indicator fluo-5N AM (10 μM, *K*_d_ = 90 μM, Invitrogen) for 45 min. Cytoplasmic dye was then washed out by obtaining whole cell recordings with Ca^2+^- and dye-free pipette solutions (Solovyova and Verkhratsky, 2002).

### VISUALIZING pHrodo-LOADED VESICLES AND MONITORING Ca^2+^ CHANGES BY TIRFM

561- and 488-nm solid-state lasers (Melles Griot, Carlsbad, CA, USA) were used to illuminate pHrodo-loaded vesicles and Ca^2+^ indicator dyes, respectively. The beam was focused off-axis onto the back focal plane of a 1.65-NA objective (Apo 100× oil, Olympus, Japan). After leaving the objective, light traveled through a high refractive index (1.78) immersion fluid (Cargille Laboratories, Cedar Grove, NJ, USA) and entered the cover slip, undergoing total internal reflection at the interface between the glass and lower refractive index of the cell membrane and overlying aqueous medium. The evanescent wave propagated at this interface declined with a length constant of 64 nm with the 561-nm laser and 57 nm with the 488-nm laser (Chen et al., 2013). For pHrodo, fluorescence emission was filtered by 609-nm (54 nm wide) bandpass filters (Semrock Inc., Rochester, NY, USA) and collected by an EMCCD camera (Hamamatsu ImagEM, Bridgewater, NJ, USA) at 40 ms/frame with a pixel size of 80 nm. For Ca^2+^ imaging with the 488-nm laser, fluorescence emission was filtered by 525-nm (45 nm wide) bandpass filters (Semrock Inc., Rochester, NY, USA) and images were obtained at 14–31 ms/frame. Imaging data were acquired using Metamorph software (Molecular Devices, Sunnyvale, CA, USA) and analyzed with Metamorph and ImageJ 1.46 (NIH, Bethesda, MD, USA).

### CELL STIMULATION

Rods were stimulated by pressure ejection (Toohey Co., Fairfield, NJ, USA) of 50 mM KCl or 10 μM ryanodine from patch pipettes or by application of depolarizing voltage steps to voltage-clamped rods. The tip of the puffer pipette was positioned 10–20 μm away from rod terminals.

For application of depolarizing steps (-70 to -10 mV, 50 or 500 ms), whole cell recordings were obtained from isolated rods, using the rod pipette solution described above. Rods were voltage-clamped with an A-M Systems Model 2400 (Carlsborg, WA, USA) amplifier. Currents were acquired and analyzed with pClamp 8 and Digidata 1200 interface (Molecular Devices). Cells with holding currents >300 pA at -70 mV were rejected from analysis.

### ASSESSING VESICLE MOBILITY FROM FLUORESCENCE CORRELATION MEASUREMENTS

To test whether CICR increased vesicle mobility in rod terminals, we loaded a small fraction of vesicles with pHrodo and then examined rod terminals under epifluorescent illumination. We focused the microscope on the cytoplasm above the membrane to avoid possible effects of release on measured changes in fluorescence. Ryanodine (10 μM) was puffed onto rod terminals to activate CICR. We compared the average frame-to-frame correlations in intraterminal fluorescence among 23 frames in the resting state to the average correlations in the CICR-activated state during the puff.

### FLUORESCENCE RECOVERY AFTER PHOTOBLEACHING

The ER of isolated rods was labeled by incubation with 1 μM ER-tracker green (Invitrogen) for 30 min at 20°C. Terminals of rods were photobleached for 5 s by illumination with a small spot (8 μm diameter) from a 30-mW 488-nm laser. ER-tracker green was illuminated by epifluorescence and images were acquired every 3 s. To show that ER-tracker dye labeled intracellular structures, we also visualized rods using a spinning disk confocal microscope (PerkinElmer Ultraview LCI).

### STATISTICAL ANALYSIS

Statistical analysis was performed with GraphPad Prism 4 (La Jolla, CA, USA). Unless otherwise stated, results are presented as mean ± SEM and statistical significance was determined using Student’s *t* test. The criterion for statistical significance was chosen to be *p* < 0.05.

## RESULTS

### DAMAGING RIBBONS BLOCKED FAST BUT NOT SLOW RELEASE FROM RODS

**FIGURE 1:**
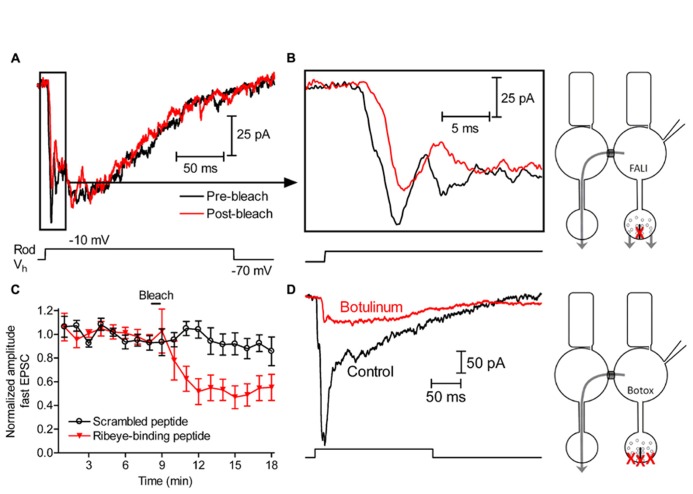
**Slow release at rod synapse is largely independent of ribbon function. (A)** EPSCs recorded from a horizontal cell voltage-clamped at -60 mV and evoked by 200-ms steps (-70 to -10 mV) applied to a rod before (black) and after (red) damaging ribbons. We show the third response obtained after fluorophore-assisted laser inactivation (FALI) with a fluorescein-conjugated Ribeye-binding peptide (80 μM). After FALI, ribbon release from the patched rod should be inhibited, but non-ribbon release from the patched rod and all of the release from neighboring rods depolarized through gap junctions should remain the same (as shown in the scheme). **(B)** To better illustrate the decrease in the initial fast component of release caused by FALI, we expanded the first 25 ms of currents shown in **(A)**. **(C)** The amplitude of the initial fast component of horizontal cell EPSCs following FALI with the Ribeye-binding peptide (red, *n* = 10 pairs) or a scrambled version of the peptide (black, *n* = 8 pairs). **(D)** Horizontal cell EPSC evoked by depolarization of a rod (-70 to -10 mV, 200 ms) immediately after obtaining a whole-cell recording (black) and after loading the rod with 500 nM botulinum toxin E through the patch pipette for ~90 min (red). Botulinum toxin E should inhibit both ribbon and non-ribbon release from the patched rod, but release from neighboring rods depolarized through gap junctions should remain the same (as shown in the scheme).

By using capacitance measurements of exocytosis in rods, we found previously that damaging the ribbon protein Ribeye by FALI significantly inhibited release evoked by short 50-ms depolarizing steps but did not significantly inhibit slow release evoked by longer 200-ms depolarizing steps (Chen et al., 2013). This suggests that ribbon release contributes to fast but not slow release from rods. To further assess the contribution of ribbons to slow release from rods, we studied the effects of damaging ribbons by FALI on glutamatergic excitatory post-synaptic currents (EPSCs) evoked in horizontal cells by depolarizing steps applied to simultaneously voltage-clamped rods (-70 to -10 mV, 200 ms; **Figure 1A**). For FALI, we used a fluorescein-conjugated peptide that binds selectively to the B domain of Ribeye (Zenisek et al., 2004). The peptide (80 μM) was introduced into rods through patch pipettes and then bleached with a 488-nm Ar/Kr laser for 50 s to cause highly localized damage to the ribbon (Snellman et al., 2011). Depolarization of rods with a short test step evokes a fast, transient EPSC (Li et al., 2010) and additional slower components are evoked by longer steps (Thoreson, 2007). Consistent with the initial fast component involving release from ribbons, we found that the amplitude of the initial fast component of the EPSC observed within the first 10 ms of the step (**Figure 1B**) was reduced by damaging the ribbon but slower components of the EPSC were not. The post-FALI response shown in **Figures 1A,B** is the third response after bleaching. The first response obtained after FALI was not diminished, consistent with results from bipolar cells and cones indicating that release of vesicles that were previously primed and attached to the ribbon was not impaired by ribbon damage (Snellman et al., 2011). As illustrated in **Figure 1C**, the fast EPSC component was reduced 49 ± 9% (*n* = 10, *p* = 0.0052) by the fourth test step after FALI. Bleaching a scrambled control version of the peptide conjugated to fluorescein did not reduce EPSCs (*n* = 5, *p* = 0.8923; **Figure 1C**).

By contrast with the reduction in fast release, damaging the ribbon by FALI did not inhibit slow release (**Figure 1A**). Comparing the charge transfer of control EPSCs to EPSCs after FALI (average of third through sixth responses), EPSCs were reduced by only 2 ± 11% (*n* = 9, *p* = 0.9342). Assuming that the 49% decline in the amplitude of the fast EPSC component means that half of the ribbon sites were damaged by FALI, this suggests that release from ribbons contributes only ~4% to the total EPSC charge transfer evoked by a 200-ms step to -10 mV.

Both staining of connexin 35/36 immunofluorescence and the ultrastructure revealed by electron microscopy show that rods in tiger salamander retina are connected to one another by gap junctions found on fin-like extensions from their somas (Zhang and Wu, 2004). Depolarizing current injected into a voltage-clamped rod can spread through these gap junctions into neighboring rods (Attwell et al., 1984; Zhang and Wu, 2005) allowing release from neighboring rods to contribute to rod-driven EPSCs (Cadetti et al., 2005). The gap junction inhibitor carbenoxolone reduced slow components of rod-driven EPSCs but causes incomplete inhibition of rod–rod coupling (Cadetti et al., 2005) and also inhibits L-type *I*_Ca_ (Vessey et al., 2004). Therefore, to measure contributions from rod–rod coupling to depolarization-evoked EPSCs, we used an alternate strategy of introducing botulinum toxin type E light chain (500 nM; R&D Systems, Minneapolis, MN, USA) into rods through the patch pipette. Botulinum toxin E cleaves the SNARE protein SNAP-25 that is expressed in photoreceptors (Ramakrishnan et al., 2012). Introduction of botulinum toxin E should therefore block vesicle fusion in the voltage-clamped rod but leave release from coupled rods unaffected (**Figure 1D**). After recording for more than an hour from rod-horizontal cell pairs with botulinum toxin in the presynaptic patch pipette, the total EPSC charge transfer was reduced by 56 ± 14% (*n* = 5, *p* = 0.0161). The portion that remained (43%) could involve SNARE-independent release (Nouvian et al., 2011) but was more likely due to spread of current into neighboring rods since this residual current was reduced another 68 ± 4% (*n* = 4, *p* = 0.0005) by hyperpolarizing neighboring rods with bright light. These results suggest that 56% of the EPSC was due to release from the voltage-clamped rod. Together with FALI results suggesting that 4% of the EPSC was due to release at ribbons, this suggests that ~7% (4%/56%) of release from voltage-clamped rods evoked by 200-ms steps to -10 mV occurred at ribbons. This supports the conclusion of other studies suggesting that rods are capable of considerable non-ribbon release (Zampighi et al., 2011; Chen et al., 2013).

### SUSTAINED DEPOLARIZATION RELEASES Ca^2+^ FROM INTRACELLULAR STORES IN ROD TERMINALS

**FIGURE 2:**
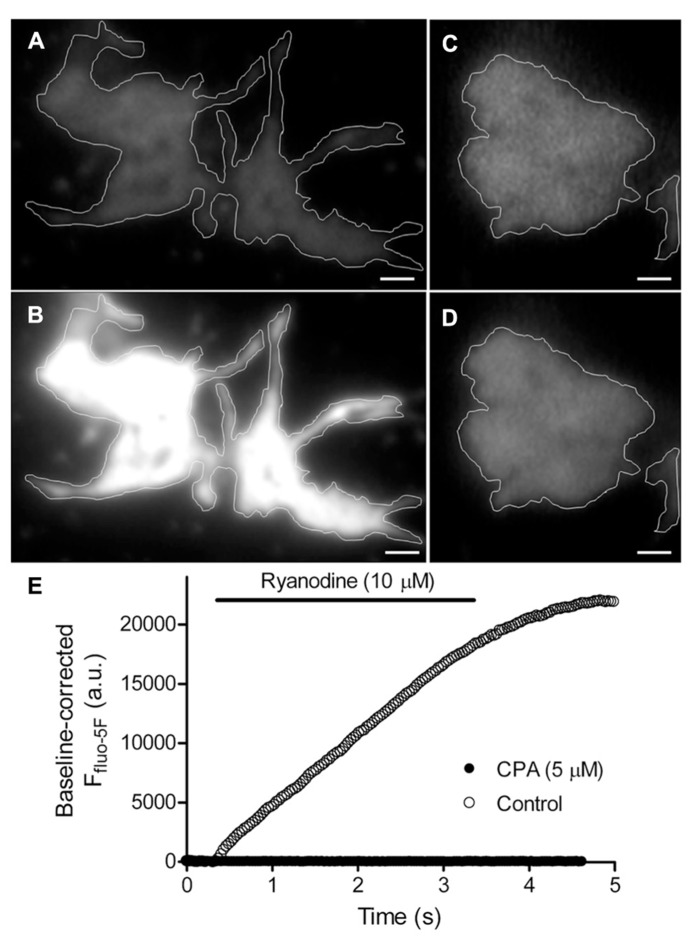
**Ca^2+^-induced Ca^2+^ release (CICR) occurs near plasma membrane in rod terminals.** Panels **(A,C)** show average images of the footprints of two rod terminals loaded with fluo-5F (averaged from 19 frames and 9 frames, respectively; 31 ms/frame). Rods were stimulated by puff application of the ryanodine receptor agonist, 10 μM ryanodine, for 3 s. In control conditions, activation of CICR with ryanodine stimulated large increase in submembrane Ca^2+^ throughout the terminal (**B**; average of 33 images). After inhibiting the sarcoplasmic/endoplasmic reticulum ATPase (SERCA) by bath application of 5 μM cyclopiazonic acid (CPA), ryanodine puff failed to stimulate a Ca^2+^ increase (**D**; average of 29 frames). The changes in fluo-5F fluorescence intensity measured within these two terminals are plotted in **(E)**. Scale bar: 1 μm.

Consistent with previous work showing that the machinery for CICR is present in rod terminals (Križaj et al., 1999, 2003; Cadetti et al., 2006), we found that activating ryanodine receptors on ER by puffing a low concentration of ryanodine (10 μM) increased submembrane [Ca^2+^]_i_ throughout rod terminals as measured by TIRFM using the Ca^2+^-sensitive dye, fluo-5F (**Figures 2A,B**; *n* = 7). **Figure 2E** shows the increase in fluo-5F fluorescence evoked by ryanodine puff measured within a region of interest in the rod terminal. After emptying ER Ca^2+^ stores by application of a sarcoplasmic/ER Ca^2+^-ATPase (SERCA) inhibitor, cyclopiazonic acid (CPA; 5 μM), ryanodine failed to stimulate an increase in [Ca^2+^]_i_ (**Figures 2C–E**, filled circles; *n* = 18). Ryanodine-evoked CICR was also blocked by pretreatment with another SERCA inhibitor, thapsigargin (1 μM; *n* = 13; not shown).

**FIGURE 3:**
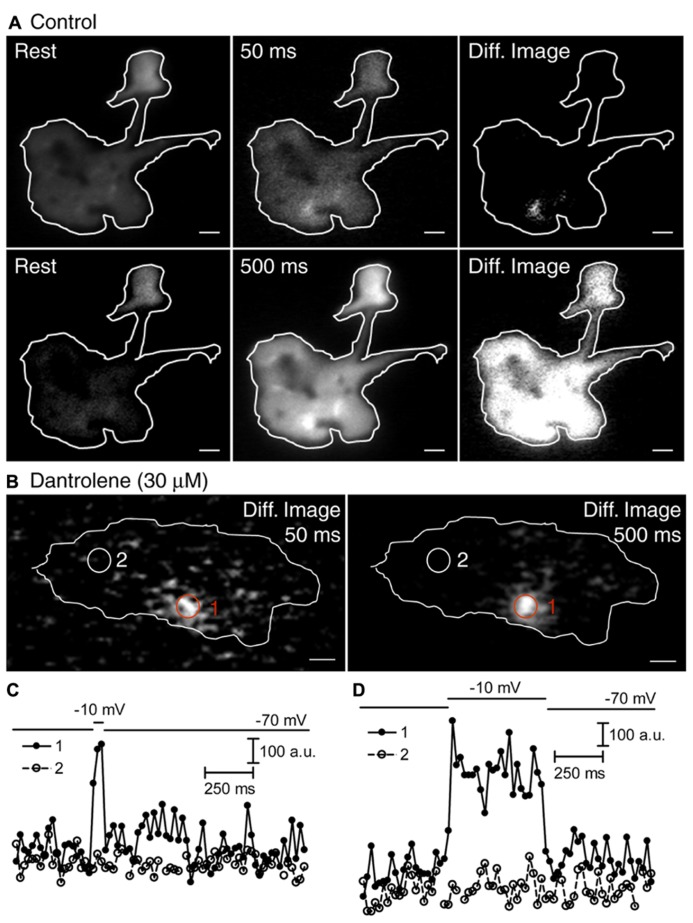
**Spread of submembrane Ca^2+^ during sustained depolarization is due to CICR.** Rods were loaded with fluo-5F (100 μM) through a patch pipette **(A,B)**. Submembrane [Ca^2+^]_i_ changes were monitored using a 488-nm laser under TIRFM at 21.3 ms/frame **(A,B)**. **(A,B)** Rods were voltage clamped and depolarized from -70 to -10 mV for 50 or 500 ms. Resting images were obtained by averaging 19 images before depolarization. Depolarized images were obtained by selecting one frame during 50-ms steps or by averaging 21 image frames during 500-ms steps. Difference images were generated by subtracting the resting images from the images during depolarization. The rod in **(A)** shows that a 50-ms depolarizing step applied under control conditions stimulated localized submembrane Ca^2+^ increases in the terminal, but a 500-ms step applied under the same conditions stimulated submembrane Ca^2+^ increases throughout the terminal. Difference images in **(B)** were generated by subtracting the resting image (average of 19 frames) before stimulation from the test image obtained during stimulation with a 50-ms step (one frame) or a 500-ms step (average of 23 frames) in the same terminal. The difference images show that Ca^2+^ increases evoked by 500-ms steps were confined after CICR was inhibited with a ryanodine receptor blocker, dantrolene (30 μM) in the patch pipette. Changes in fluo-5F fluorescence intensities within region 1 (red circles) and region 2 (white circles) were plotted against time for both 50-ms **(C)** and 500-ms steps **(D)**. Scale bar: 1 μm.

We examined the sources of Ca^2+^ involved in depolarization-evoked increases in submembrane [Ca^2+^]_i_. Consistent with earlier findings (Cadetti et al., 2006; Chen et al., 2013), 50-ms depolarizing steps (-70 to -10 mV) triggered spatially confined Ca^2+^ increases (**Figure 3A**, top row), reflecting the clustering of Ca^2+^ channels adjacent to ribbons (Nachman-Clewner et al., 1999; Tom Dieck et al., 2005). Longer 500-ms steps triggered Ca^2+^ increases that spread throughout the terminal (**Figure 3A**, bottom row). Blocking ryanodine receptors by addition of a ryanodine receptor blocker, dantrolene (30 μM, **Figure 3B**), or a high concentration of ryanodine (100 μM, not shown) to the pipette solution did not affect Ca^2+^ influx evoked by 50-ms steps (**Figure 3B**, left), but confined the spread of Ca^2+^ evoked by 500-ms depolarizing steps as expected from a block of CICR (**Figure 3B**, right). The time course for Ca^2+^ changes in two regions of interest (**Figures 3C,D**), placed over a focal Ca^2+^ entry site (region 1; **Figure 3B**) and away from focal Ca^2+^ entry sites (region 2; **Figure 3B**), further confirmed the effectiveness of the ryanodine receptor-mediated block of CICR during sustained depolarization. After blocking CICR, depolarization with a 500-ms step evoked a localized Ca^2+^ increase in region 1 near the presumed location of a ribbon but evinced little change in other parts of the terminal (e.g., region 2). Thus, blocking CICR converted the [Ca^2+^]_i_ response evoked by a sustained depolarization to a confined response typically observed following brief depolarizing steps.

Similar to these voltage-clamp experiments, depolarization evoked by puffing high KCl solutions (50 mM KCl; 500 ms to 1 s) increased [Ca^2+^]_i_ throughout the terminal and this spread of Ca^2+^ was confined by blocking CICR with dantrolene (10 μM, not shown). These findings show that the spread of submembrane Ca^2+^ during sustained depolarization is due to CICR, consistent with previous studies (Križaj et al., 1999, 2003; Cadetti et al., 2006; Babai et al., 2010b).

### Ca^2+^ RELEASED FROM INTRACELLULAR STORES TRIGGERS SYNAPTIC VESICLE RELEASE

**FIGURE 4:**
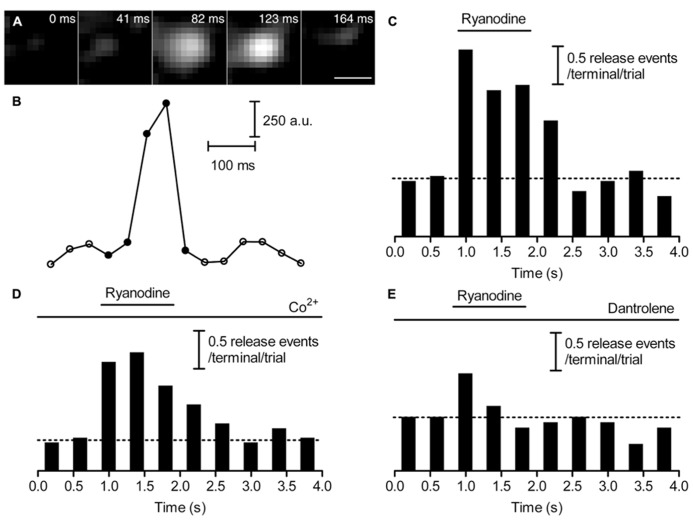
**Ca^2+^ released from intracellular stores is capable of triggering release. (A)** Sequential images show a pHrodo-loaded vesicle brightening as it approached the membrane and then disappearing due to fusion, following stimulation with a puff of ryanodine (10 μM). Scale bar: 500 nm. **(B)** Changes in fluorescence intensity within a 560-nm diameter region of interest placed over this vesicle are plotted against time in **(B)**. Filled circles indicate the fluorescence intensities of the five frames shown in **(A)**. To plot release kinetics in panels **(C–E)**, we counted the number of synaptic vesicle release events per rod terminal per trial, binned events in 40-ms increments, and plotted them against time. **(C)** In control conditions, the number of release events increased during puff application of ryanodine (10 μM; *n* = 15 rods). **(D)** Ryano-dine (10 μM; *n* = 16 rods) stimulated an increase in release when rods were superfused with Ca^2+^-free Ringer’s solution containing Co^2^^+^ (1 mM, *n* = 16 rods). **(E)** Ryanodine (10 μM; *n* = 14 rods) caused little increase in release in the presence of a ryanodine receptor blocker, dantrolene (10 μM). The dashed line in **(C–E)** shows the level of release before ryanodine puff.

The finding that Ca^2+^ released from ER can attain high levels just beneath the membrane suggests that CICR may be able to stimulate synaptic vesicle release. To visualize CICR-mediated individual synaptic vesicle release, we loaded a small percentage of synaptic vesicles in rod terminals with dextran-conjugated pHrodo (Chen et al., 2013) and transiently exposed them to an agonist concentration of ryanodine (10 μM). During ryanodine puffs, fluorescently labeled vesicles brightened as they approached the membrane and disappeared rapidly as they fused (**Figures 4A,B**). By counting the number of release events at each time point, we found that vesicle release increased during stimulation of CICR with ryanodine (10 μM, **Figure 4C**). Co^2^^+^ (1 mM) in a nominally Ca^2+^-free solution did not inhibit ryanodine-evoked release (**Figure 4D**) although this same condition blocked release evoked by puff application of 50 mM KCl (Chen et al., 2013). However, co-application of dantrolene (10 μM, **Figure 4E**) inhibited vesicle release during ryanodine puffs. Thus, CICR can trigger release even when voltage-operated Ca^2+^ channels are blocked.

At *Drosophila* neuromuscular junction, CICR enhances release by increasing vesicle mobility (Shakiryanova et al., 2005). To test whether CICR increased vesicle mobility in rod terminals, we puffed ryanodine (10 μM) onto terminals and analyzed frame-to-frame fluorescence correlations to assess changes in vesicle mobility. We focused on cytoplasmic vesicles in the center of the terminal illuminated by epifluorescence, not TIRFM. Activation of CICR did not alter fluorescence correlations indicating that CICR did not alter vesicle mobility (correlation coefficients at rest = 0.910 and during ryanodine puff application = 0.907, *n* = 8, *p* = 0.47, paired *t*-test). This is consistent with results of fluorescence recovery after photobleach (FRAP) experiments showing that unlike conventional synapses where most vesicles are tethered to actin via synapsin (Benfenati et al., 1989), vesicles in ribbon synapses are largely mobile and their mobility is unaffected by [Ca^2+^]_i_ (Holt et al., 2004; Rea et al., 2004; Chen et al., 2013).

### CICR EVOKED NON-RIBBON RELEASE DURING SUSTAINED DEPOLARIZATION

**FIGURE 5:**
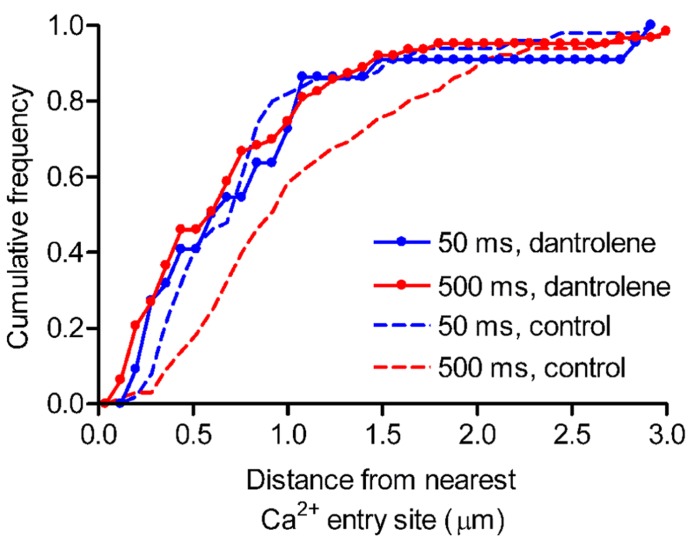
**Inhibiting CICR with dantrolene decreased non-ribbon release.** Rods were loaded with pHrodo to label synaptic vesicles and then voltage clamped. Fluo-5F (100 μM) and dantrolene (30 μM) were introduced through the patch pipette. Rods were stimulated with 50- or 500-ms steps from -70 to -10 mV. The relative cumulative frequencies of pHrodo-loaded vesicle release events stimulated by 50- (blue circles, *n* = 22 events from nine rods) or 500-ms (red circles, *n* = 63 events from nine rods) steps plotted against distance from the nearest Ca^2+^ entry site. For comparison, the cumulative frequencies of release events obtained under control conditions without dantrolene are replotted from Chen et al. (2013) for 50- (blue dashed line) and 500-ms (red dashed line) steps. When CICR was inhibited with dantrolene, release events triggered by 500-ms steps clustered close to Ca^2+^ entry sites, matching the distribution seen with release evoked by 50-ms steps [Kolmogorov–Smirnov (K–S) test: *p* = 0.936]. By contrast, the distribution of release events evoked by 500-ms steps in control conditions differed significantly from the distribution of release events evoked by 500-ms steps in the presence of dantrolene (K–S test: *p* = 0.002). Scale bar: 1 μm.

To investigate the spatial distribution of release events triggered by Ca^2+^ from intracellular stores, we mapped individual vesicle release events. Focal Ca^2+^ entry sites visualized during 50-ms depolarizing steps (**Figure 3A**) were used to identify ribbon locations. The distance from the nearest Ca^2+^ entry site was measured for each release event. Previously (Chen et al., 2013), we found that 500-ms steps evoked greater non-ribbon release than 50-ms steps. To test whether additional non-ribbon release during 500-ms steps was due to CICR-mediated spread of Ca^2+^ throughout the terminal, we included dantrolene (30 μM) in the pipette solution. As described above, inclusion of dantrolene blocked CICR and resulted in a confined [Ca^2+^] increase during 500-ms steps that resembled confined [Ca^2+^] increases triggered by 50-ms depolarizing steps (**Figure 3B**). Dantrolene also confined release events evoked by 500-ms steps close to focal Ca^2+^ entry sites (**Figure 5**). In the presence of dantrolene, most (~75%) of the release events evoked by 500-ms steps occurred within 1 μm of the focal Ca^2+^ entry sites, matching the distribution of release events evoked by 50-ms steps in the presence or absence of dantrolene (**Figure 5**). For comparison, salamander rod ribbons average ~1 μm in length (Townes-Anderson et al., 1985). Data from Chen et al. (2013) were re-plotted in **Figure 5** (dashed red line) and show that 500-ms steps applied in control conditions evoked a significantly larger number of release events than 50 ms steps (~50%, Kolmogorov–Smirnov test: *p* = 0.019) > 1 μm from the ribbon. Consistent with electrophysiological results showing the contribution of CICR to slow release (Cadetti et al., 2006; Suryanarayanan and Slaughter, 2006), dantrolene also strongly inhibited slower components of release evoked by 500-ms steps measured optically (data not shown). These results indicate that virtually all of the additional non-ribbon release evoked by sustained depolarization is due to CICR.

**FIGURE 6:**
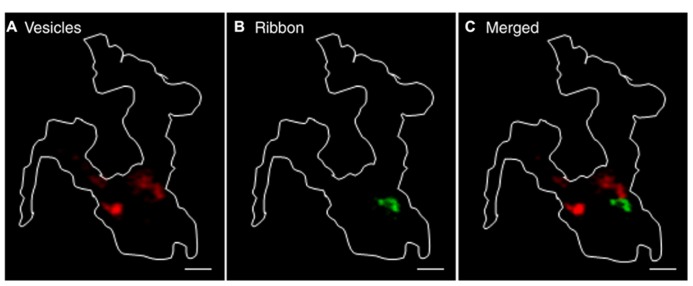
**Ca^2+^ released from intracellular stores evokes non-ribbon release.** A large number of synaptic vesicles in rods were loaded with pHrodo by 30-min incubation. Rods were voltage clamped and loaded with fluo-5F through the pipette. **(A)** Vesicle recruitment map in a rod terminal that was puffed with 10 μM ryanodine. The map was generated by subtracting the average of 37 resting images before puff from the average of 52 test images during puff. **(B)** Ca^2+^ entry sites visualized by depolarizing the same rods with a 50-ms step from -70 to -10 mV to trigger influx through Ca^2+^ channels clustered near ribbons. The difference image was generated by subtracting the average of 15 resting images before the step from an average of two test images during the 50-ms depolarizing test step. The overlaid image in **(C)** shows that sites of vesicle recruitment and release did not overlap with locations of ribbons shown by focal Ca^2+^ entry sites. Scale bar: 1 μm.

To observe the behavior of a large number of vesicles, we incubated rods with pHrodo for 30 min. We mapped sites of vesicle recruitment and subsequent release from increases in near-membrane fluorescence that accompanied membrane approach of vesicles just prior to fusion. Since 85% of near-membrane vesicles subsequently fuse, recruitment maps provide a good map of release sites (Chen et al., 2013). CICR was activated by ryanodine (10 μM) puff application. Recruitment maps were generated by subtracting the average control image from the average stimulated image obtained during ryanodine puff. A representative recruitment map is shown in **Figure 6A**. To map ribbon locations, we voltage-clamped these rods and loaded them with fluo-5F to visualize focal sites of Ca^2+^ entry evoked by depolarization with 50-ms steps (e.g., **Figure 6B**). As illustrated by overlay of a vesicle recruitment map (red) and ribbon locations (green) in **Figure 6C**, the sites to which vesicles were recruited by activation of CICR did not overlap with ribbons in five out of six rods. In one rod, there was partial overlap. Given that release-ready vesicles are thought to cluster primarily at ribbons, it was surprising to find that direct stimulation of CICR stimulated recruitment and subsequent release almost exclusively at non-ribbon sites. However, the finding that CICR triggers release almost entirely at non-ribbon sites complements the previous finding that non-ribbon release during sustained depolarization is almost entirely due to CICR.

**FIGURE 7:**
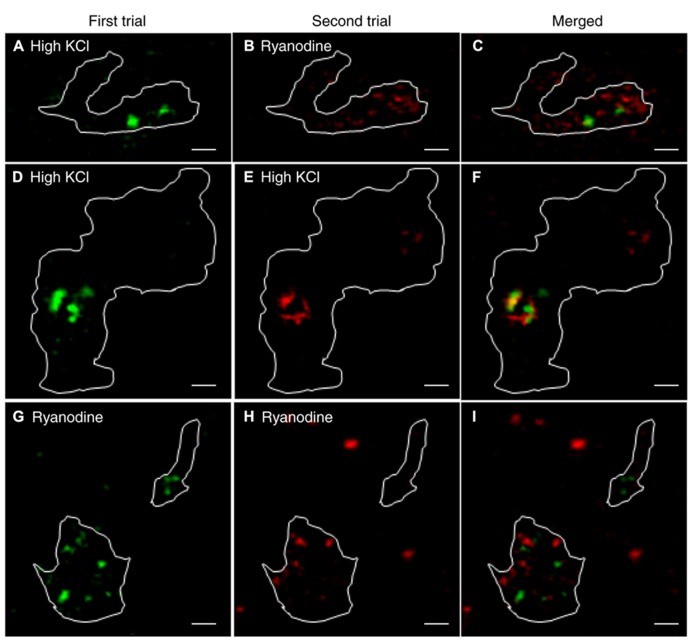
**Release evoked by depolarization occurred at fixed ribbon sites whereas non-ribbon release evoked by ryanodine occurred at locations that are not fixed.** A large number of vesicles were labeled by incubating rods with pHrodo for 30 min. To show the location of vesicles recruited to the membrane for release, we generated difference images by subtracting the average of resting images before stimulation from test images obtained during stimulation in the same rod terminal. Panel **(A)** shows the recruitment map for a rod terminal puffed with 50 mM KCl for 1 s. Panel **(B)** shows the same terminal puffed with 10 μM ryanodine for 1 s. The overlay of **(A,B)** in panel **(C)** shows that KCl and ryanodine stimulated release at largely different locations. **(D–F)** Stimulating a rod terminal twice in succession with 500 ms puffs of 15 mM KCl evoked release at similar locations in both trials (**D**: first puff; **E**: second puff; **F**: merged; *n* = 7). **(G–I)** Stimulating a rod terminal twice in succession with 1 s puffs of 1 μM ryanodine evoked release at different locations in both trials (**G**: first puff; **H**: second puff; **I**: merged; *n* = 6). Scale bar: 1 μm.

We also compared the location of release sites evoked by activation of CICR with ryanodine (10 μM) to those evoked by depolarizing stimulation with 50 mM KCl in the same terminals. Recruitment maps were generated in terminals loaded by 30-min incubation with pHrodo as described above. Vesicle recruitment triggered by puff application of 50 mM KCl was concentrated at a few sites in each terminal with some additional recruitment observed elsewhere in the terminal (**Figure 7A**). The clustering of newly recruited vesicles observed during depolarizing stimulation co-localizes with synaptic ribbons (Chen et al., 2013). These results suggest that depolarizing stimulation with a high concentration of K^+^ triggers ribbon release as well as some additional release at non-ribbon sites. Ribbon release was presumably triggered by Ca^2+^ influx through Ca^2+^ channels clustered near the ribbon whereas non-ribbon release was due to the secondary activation of CICR.

Release of Ca^2+^ from intracellular stores evoked by puff application of ryanodine (10 μM) stimulated vesicle recruitment and release at more diffusely distributed locations throughout the terminal than high K^+^ stimulation (**Figure 7B**). Furthermore, sites of vesicle recruitment triggered by activation of CICR with ryanodine differed from sites of vesicle recruitment and release triggered by depolarizing stimulation with 50 mM KCl (**Figure 7C**; *n* = 5 rods). Because depolarizing stimulation preferentially stimulated release from ribbon sites, this provides further evidence that CICR preferentially stimulates release at non-ribbon sites.

To test whether non-ribbon release occurs at fixed locations, we repeatedly stimulated CICR in rod terminals with puff application of ryanodine. We also tested repeated stimulation by puff application of high KCl solutions. To keep cells healthy and improve responses to repeated stimulation, the stimulus strength was reduced (1 μM ryanodine puff for 1 s to activate CICR; 15 mM KCl for 500 ms as a depolarizing stimulus). Puff application of KCl twice in succession triggered vesicle recruitment and release concentrated at overlapping locations in both trials although there was also some vesicle recruitment to other sites (**Figures 7D–F**; *n* = 7 rods). Vesicle recruitment at these additional sites may be due to activation of CICR by KCl puffs. By contrast with the largely repeatable sites evoked by KCl puffs, two successive puffs of ryanodine triggered vesicle recruitment and release at different sites in the two trials (**Figures 7G–I**; *n* = 6 rods). These results suggest that depolarization-evoked release at ribbons occurs at fixed locations whereas sites of non-ribbon release triggered by CICR are not fixed.

### Ca^2+^ CAN DIFFUSE THROUGH CONTINUOUS ER EXTENDING FROM SOMA TO TERMINAL

If sustained release requires CICR, then ER Ca^2+^ stores must be capable of maintaining CICR during sustained depolarization. Consistent with this, we found that submembrane Ca^2+^ levels remained elevated throughout rod terminals when depolarized by 15 mM KCl for 10 s (*n* = 9). Furthermore, evidence that blocking CICR inhibits light responses in second-order neurons and ongoing glutamate release from rods suggests that CICR can be maintained almost indefinitely (Cadetti et al., 2006; Suryanarayanan and Slaughter, 2006; Babai et al., 2010b). These results prompted us to ask how CICR can be maintained for long periods of time.

**FIGURE 8:**
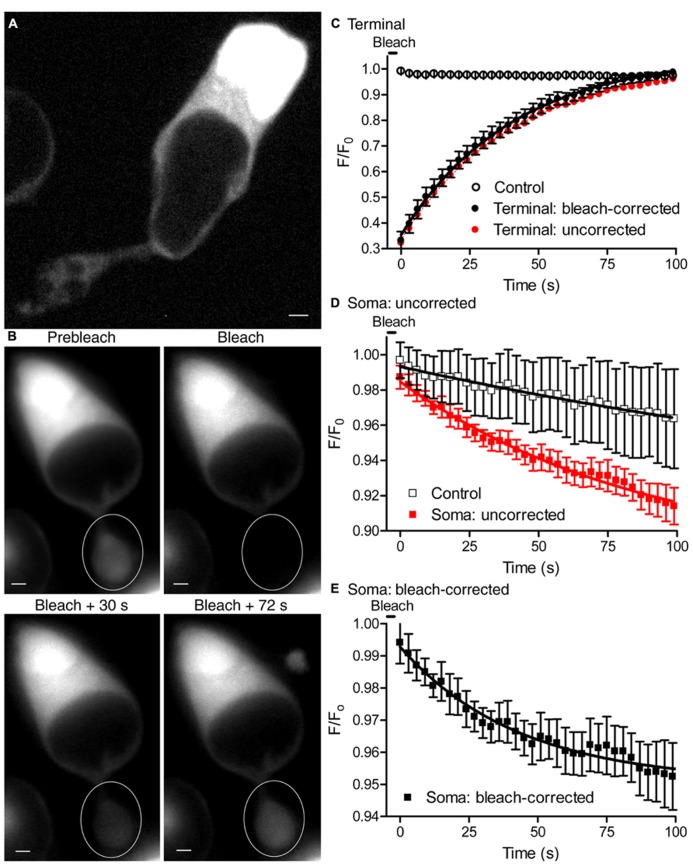
**Endoplasmic reticulum is continuous from soma to terminal. (A)** A single confocal plane of an isolated rod labeled with ER-tracker green (1 μM) and imaged with a spinning disk confocal microscope. Labeled intracellular structures are consistent with the presence of ER throughout the soma and terminal. **(B)** Series of images from an isolated rod loaded with ER-tracker green, showing fluorescence before and after laser photobleaching of the synaptic terminal (white circle). Scale bar: 2 μm. **(C)** The time course of fluorescence (*F*/*F*_0_) changes in terminals after photobleaching with 488-nm laser spot of 8 μm in diameter (filled red circles). *F*_0_ is the average fluorescence intensity in the terminal before bleaching. Fluorescence changes in control terminals that were not laser photobleached are also plotted (*n* = 5, open circles) to show bleaching caused by the epifluorescence illumination used to measure fluorescence recovery. The time course of fluorescence changes in terminals that were laser photobleached are also plotted after subtracting the bleaching caused by epifluorescent measurements (filled black circles). The solid black line shows an exponential fit to the fluorescence recovery in bleach-corrected terminals (τ = 38 s, *n* = 6 terminals). Photobleaching was triggered at time 0 and recovery was measured every 3 s. **(D)** Time course of *F*/*F*_0_ measured in the soma. Red squares show fluorescence changes in the somas of rods whose terminals were photobleached (τ = 90 s, *n* = 6). Black open squares show fluorescence changes in the somas of control rods whose terminals were not photobleached (τ = 278 s, *n* = 6). The small, slow fluorescence decline observed in somas of control rods whose terminals were not photobleached reflects the bleaching caused by epifluorescent measurements. **(E)** The rate of fluorescence decline in somas of rods with photobleached terminals after correcting for bleaching induced by epifluorescent measurements (*t* = 43 s; *n* = 6).

Ultrastructural studies of photoreceptors show the presence of ER in the terminal, axon, and soma (Mercurio and Holtzman, 1982). In other polarized cells, ER forms a continuous network throughout the cell allowing Ca^2+^ ions to tunnel through ER from one region to another (Mogami et al., 1997; Choi et al., 2006; Petersen and Verkhratsky, 2007). We hypothesized that the ER network in rods may form a continuous network allowing Ca^2+^ to diffuse from soma to terminal ER in support of sustained synaptic release. To test whether ER in rods forms a continuous structure extending from terminal to soma, we labeled ER with the dye ER-tracker green, which binds to sulphonylurea receptors of ATP-sensitive K^+^ channels that are prominent on ER (Hambrock et al., 2002). Confocal images of isolated rods labeled with ER-tracker green showed fluorescent labeling of intracellular structures, presumably ER, throughout the soma and terminal (**Figure 8A**). To test for continuity of these labeled structures, we measured recovery of terminal fluorescence after photobleaching ER-tracker green with a laser spot (488 nm, 30 mW, 8 μm in diameter) centered on the terminal. We monitored cell fluorescence at 3 s intervals before and after laser photobleaching (**Figure 8B**). By measuring the rate of fluorescence decline in terminals and somas of control cells from the same microscope field that were not photobleached by the laser, we determined that epifluorescent measurements made every 3 s caused a small degree of bleaching (<4% in 100 s; **Figure 8**), independent of laser photobleach (65%). Epifluorescent measurement caused similar bleaching in terminals (**Figure 8C**, open circles) and somas (**Figure 8D**, open squares) of control cells. After subtracting the bleaching caused by epifluorescent measurements, the rate of FRAP by the laser exhibited a time constant (τ) of 38.3 s (**Figure 8C**, filled black circles) Without correcting τ for bleaching by the measurement light, terminal fluorescence recovered with τ = 38.8 s (**Figure 8C**, filled red circles). Recovery of terminal fluorescence is due to dye molecules diffusing to the terminal from other parts of the cell, including the soma. We therefore measured fluorescence changes in the somas of cells whose terminals were photobleached. Before correcting for bleaching by the epifluorescent measurement light, the fluorescence intensity declined after laser photobleach more rapidly in somas of cells whose terminals were photobleached (**Figure 8D**, filled red squares) than in somas of control cells whose terminals were not photobleached (**Figure 8D**, open black squares). After correcting for bleaching by epifluorescent measurement, the fluorescence signal in the soma declined exponentially with τ = 43.1 s, close to the rate of FRAP in the terminal (**Figure 8E**). The amount of fluorescence decline in the soma caused by laser photobleach was much smaller than the amount of fluorescence recovered in the terminal, as expected from the much larger volume of ER in the soma. These results suggest that dye can diffuse from soma to terminal, restoring fluorescence in the terminal and slightly diminishing fluorescence in the soma.

The rate of fluorescence recovery in the terminal was consistent with the expected rate of diffusion of ER-tracker dye through the ER. The diffusion coefficient of IP_3_R_1_ receptors on ER membranes is 0.3 μm^2^/s (Fukatsu et al., 2004) and ether-a-go-go (EAG) K^+^ channels on the plasma membrane exhibit diffusion coefficients of 0.1–0.3 μm^2^/s (Gómez-Varela et al., 2010). Assuming χ^2^ = 2Dt and a diffusion coefficient of 0.3 μm^2^/s for ATP-sensitive K^+^ channels in ER, then channels would move an average of ~5 μm in 38 s, similar to the distance from soma to terminal. Thus, results of FRAP experiments indicate that the ER forms a continuous structure extending from the terminal to soma in rods.

**FIGURE 9:**
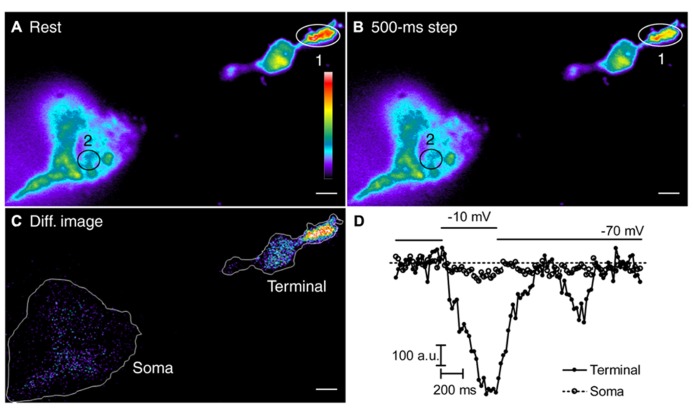
**Sustained depolarization depletes Ca^2+^ from intracellular stores in the terminal more than the soma. (A,B)** The rod was loaded with a low affinity Ca^2+^ indicator, fluo-5N AM and then patched with a pipette containing Ca^2+^- and dye-free solutions to wash dye out of the cytoplasm. Rods were depolarized for 500 ms from -70 to -10 mV. Changes in ER [Ca^2+^] were monitored using TIRFM at 22 ms/frame. **(A)** The resting pseudocolor image is the average of 20 frames before stimulation. **(B)** The depolarized pseudocolor image is the average of 15 frames during stimulation. **(C)** The difference pseudocolor image was obtained by subtracting the depolarized image from the resting image. In this difference image, hotter colors show larger declines in ER [Ca^2+^]. **(D)** The fluorescence intensities of fluo-5N in the terminal (region 1, filled circles) and soma (region 2, open circles) were plotted against time. Scale bar: 2 μm.

To visualize ER Ca^2+^ levels of 60–400 μM (Michalak et al., 2002), we loaded rods with a low affinity Ca^2+^ dye, fluo-5N AM (*K*_d_ = 90 μM). We then obtained whole cell patch clamp recordings using a Ca^2+^- and dye-free pipette solution to wash dye out of the cytoplasm (Solovyova and Verkhratsky, 2002). Many brightly fluorescent areas were visible within terminals by TIRFM (**Figure 9A**; *n* = 6 rods), consistent with that the presence of ER Ca^2+^ stores close to the membrane. We depolarized rods with 500-ms steps to test whether these bright areas represented functional intracellular Ca^2+^ stores mediating CICR. As illustrated in **Figure 9**, ER [Ca^2+^] decreased in the terminal during the depolarizing step and recovered soon thereafter. This confirms that sustained depolarization releases Ca^2+^ from intracellular Ca^2+^ stores in rod terminals. Application of a 500-ms depolarizing step caused only a very small decrease in soma ER [Ca^2+^]. Similar results were seen in four cells. The decrease in soma ER [Ca^2+^] could be due to diffusion of Ca^2+^ through the ER from soma to terminal, although it might also be due to release of Ca^2+^ into the soma cytoplasm. Regardless, this result shows that release of Ca^2+^ from terminal ER did not significantly deplete Ca^2+^ from soma ER during a 500-ms step. Thus, soma ER Ca^2+^ stores appear to have the capacity to sustain the transfer of Ca^2+^ from cell body to terminal during sustained depolarization.

## DISCUSSION

### CICR TRIGGERS RELEASE AT NON-RIBBON SITES

Rather than simply facilitating release as found at a number of other synapses (Bouchard et al., 2003), we found that CICR directly triggers release at non-ribbon sites. The ability of CICR to trigger release directly is partly due to the ability of submicromolar [Ca^2+^] to stimulate release from rods, much lower than the [Ca^2+^] needed to stimulate release from other neurons (Rieke and Schwartz, 1996; Thoreson et al., 2004; Duncan et al., 2010). The principal ryanodine receptor subtype in rods is a variant of RyR2 (Shoshan-Barmatz et al., 2007). The probability of RyR2 opening can be regulated not only by local Ca^2+^ levels but also by phosphorylation, redox state, FK506-binding proteins, and other mechanisms (Zalk et al., 2007) suggesting that these factors may also be capable of regulating release.

Consistent with ultrastructural evidence of ER close to the plasma membrane in rod terminals (Mercurio and Holtzman, 1982; Babai et al., 2010b), activation of CICR stimulated submembrane Ca^2+^ increases throughout the terminal. Despite the fact that release-competent vesicles are concentrated near ribbons (Zenisek, 2008; Frank et al., 2010; Snellman et al., 2011), ryanodine triggered release almost exclusively at non-ribbon sites. The ineffectiveness of CICR in triggering release from ribbons may be due to a barrier that limits diffusion of Ca^2+^ or mobile Ca^2+^ buffers to and from ribbon release sites. Such a barrier was suggested by electrophysiological experiments with diffusible buffers (Bartoletti et al., 2011). Another possibility may be that the molecular mechanisms mediating exocytosis at non-ribbon sites differ from those mediating release at ribbon sites (e.g., by use of a different, higher affinity Ca^2+^ sensor).

Activation of CICR by repeated application of low concentrations of ryanodine triggered release at non-ribbon sites that varied in location from trial to trial. On the other hand, depolarization with KCl puffs, which stimulates release preferentially at ribbons (Chen et al., 2013), triggered release largely at fixed, reproducible locations. One possible interpretation of these data is that non-ribbon release sites are not fixed, but mobile. Perhaps release at non-ribbon sites involves target-SNAREs (t-SNAREs) that wander freely about the rod terminal membrane after escaping endocytosis in perisynaptic regions (Wahl et al., 2013). However, non-ribbon release in bipolar cells occurs adjacent to relatively fixed post-synaptic densities (Midorikawa et al., 2007). Another possibility is that non-ribbon release in rods may also occur at fixed sites but with such low release probability that release rarely recurs at the same site in repeated trials.

### RELEASE AT PHOTORECEPTOR SYNAPSES

Much of our current understanding of the mechanisms of release from photoreceptors is based on studies at cone synapses. In cones, the immediately releasable pool (IRP) of vesicles tethered at the base of the ribbon is replenished when they remain hyperpolarized in bright light. Subsequent membrane depolarization accompanying a decrement in light triggers the opening of L-type Ca^2+^ channels beneath the ribbon and stimulates the rapid release of vesicles (Bartoletti et al., 2010; Snellman et al., 2011). Release of a vesicle from the IRP in cones can be stimulated by the opening of <3 Ca^2+^ channels (Bartoletti et al., 2011). During maintained depolarization in darkness, the IRP is soon emptied (Jackman et al., 2009). After this pool has been emptied, the sustained release of vesicles by cones is no longer regulated by individual Ca^2+^ channel openings but is instead governed by the Ca^2+^-dependent delivery of release-competent vesicles to release sites at the base of the ribbon (Jackman et al., 2009; Babai et al., 2010a).

Li et al. (2010) showed that brief 1 ms test steps applied to ground squirrel rods can evoke fast EPSCs but did not report the effects of longer test steps. In amphibian retina, use of longer test steps evoke an initial burst of fast release followed by pronounced slow release (Cadetti et al., 2005; Rabl et al., 2005; Thoreson, 2007). Damaging rod ribbons by FALI diminished fast release measured both by paired recordings (**Figure 1**) and by capacitance techniques (Chen et al., 2013) indicating that the initial burst of release from rods involves the ribbon. Like cones, it is therefore also likely that some of the slow release from rods involves replenishment of ribbon-release sites. However, damaging rod ribbons did not significantly inhibit slow release suggesting that much of the slow release involves non-ribbon sites. Slow release is amplified by CICR in both amphibian and mammalian rods (Cadetti et al., 2006; Suryanarayanan and Slaughter, 2006; Babai et al., 2010b) and the present results showed that CICR promotes slow release by triggering vesicle fusion at non-ribbon sites. For example, when rods were stimulated with 500-ms depolarizing steps to activate CICR, ~50% of release events occurred >1 μm from the ribbon (Chen et al., 2013) and the present results showed that virtually all of the non-ribbon release events evoked by 500-ms steps were triggered by CICR (**Figure 5**). The evidence for substantial non-ribbon release from rods in retinal slices obtained by FALI experiments suggests that non-ribbon release observed by TIRFM in isolated rods is not an artifact of cell isolation procedures.

Consistent with a significant role for non-ribbon release from rods, electron tomography studies revealed frequent exocytotic omega figures in non-ribbon regions of rod synapses (Zampighi et al., 2011). In addition, the absence of anchored ribbons in Bassoon mutant mice did not abolish visually guided behavior, visual cortical responses, or bipolar cell light responses assessed with the electroretinogram b-wave (Dick et al., 2003; Goetze et al., 2010). However, it is possible that the residual release in Bassoon mutants originates from a small number of intact ribbon synapses or release sites that contain the normal molecular machinery and would have been occupied by a ribbon (Dick et al., 2003). In support of a substantial role for CICR-triggered non-ribbon release, blocking CICR inhibited light responses of second-order neurons in amphibian retina by as much as 90% (Cadetti et al., 2006; Suryanarayanan and Slaughter, 2006) and in mouse retina by ~50% (Babai et al., 2010b).

The extent of non-ribbon release at other ribbon synapses is not clear. Release from Ca^2+^ stores has been implicated in driving sustained release from hair cells (Schnee et al., 2011). However, dissociating ribbons from the membrane by mutating the active zone protein bassoon decreased both fast and sustained release from hair cells (Khimich et al., 2005; Frank et al., 2010). Damaging ribbons by FALI reduced fast and sustained release equally in both cones and bipolar cells (Snellman et al., 2011), although TIRFM experiments also suggest a role for non-ribbon release in bipolar cells (Zenisek et al., 2004; Midorikawa et al., 2007; Zenisek, 2008).

### ER FACILITATES SUSTAINED RELEASE BY TRANSFERRING Ca^2+^ FROM SOMA TO TERMINAL

The activation of CICR during sustained depolarization appears to provide supplemental Ca^2+^ that is essential for sustaining release from rods in darkness. This may be particularly important at rod synapses where the continued influx of Ca^2+^ during sustained depolarization can cause extracellular [Ca^2+^] to decline significantly in the synaptic cleft (Rabl et al., 2003). This decline reduces the amplitude of *I*_Ca_ and thereby reduces sustained release.

Results from other polarized cells show that Ca^2+^ ions can “tunnel” through the ER from distant parts of the cell (Mogami et al., 1997; Park et al., 2000; Choi et al., 2006; Petersen and Verkhratsky, 2007). FRAP experiments with ER-tracker dye showed that the ER forms a continuous structure in rods, extending from terminal to soma. Along with ellipsoid mitochondria, ER in the cell body can store large amounts of Ca^2+^ (Michalak et al., 2002; Szikra and Krizaj, 2007). Consistent with this, 500-ms depolarizing steps depleted Ca^2+^ from terminal ER, but caused barely detectable reductions in soma ER. Furthermore, we found that CICR could be sustained in rod terminals by depolarizing stimuli lasting for 10 s. Together, these results support the idea that Ca^2+^ may sustain CICR by tunneling through the ER from more distant reservoirs. As Ca^2+^ diffuses through the ER to the terminal to replenish ions depleted by CICR, Ca^2+^ can be restored to the ER in other parts of the cell by the actions of sarcoplasmic/ER Ca^2+^-ATPase type 2 (SERCA2) which pumps Ca^2+^ from the cytoplasm (Križaj, 2005). Store-operated Ca^2+^ entry also helps to regulate release from rods by elevating cytoplasmic Ca^2+^ and replenishing ER Ca^2+^ (Szikra et al., 2008; García-Sancho, 2014). Together, these non-conventional mechanisms appear to sustain CICR and may thereby sustain non-ribbon release in darkness.

### FUNCTIONAL IMPLICATIONS

By triggering release at non-ribbon sites, CICR increases the total amount of slow release from rods thereby amplifying the rate of release in darkness. Elevated release in darkness enhances decrements in release that occur when rods hyperpolarize to light. Use of distant non-ribbon release sites may also have a secondary benefit of reducing synaptic noise by diffusional filtering of glutamate to post-synaptic glutamate receptors (DeVries et al., 2006). Together, these effects of CICR-mediated release at non-ribbon sites may improve the signal-to-noise ratio, enhancing contrast discrimination and the detection of small responses near visual threshold (Pahlberg and Sampath, 2011). Overall, the present results indicate that the triggering of release at non-ribbon sites by CICR is a major mechanism by which rods maintain the release of vesicles in darkness.

## Conflict of Interest Statement

The authors declare that the research was conducted in the absence of any commercial or financial relationships that could be construed as a potential conflict of interest.
